# Favipiravir (T-705) Inhibits Junín Virus Infection and Reduces Mortality in a Guinea Pig Model of Argentine Hemorrhagic Fever

**DOI:** 10.1371/journal.pntd.0002614

**Published:** 2013-12-26

**Authors:** Brian B. Gowen, Terry L. Juelich, Eric J. Sefing, Trevor Brasel, Jennifer K. Smith, Lihong Zhang, Bersabeh Tigabu, Terence E. Hill, Tatyana Yun, Colette Pietzsch, Yousuke Furuta, Alexander N. Freiberg

**Affiliations:** 1 Department of Animal, Dairy, and Veterinary Sciences, Utah State University, Logan, Utah, United States of America; 2 Institute for Antiviral Research, Utah State University, Logan, Utah, United States of America; 3 School of Veterinary Medicine, Utah State University, Logan, Utah, United States of America; 4 Department of Pathology, The University of Texas Medical Branch, Galveston, Texas, United States of America; 5 Department of Microbiology and Immunology, The University of Texas Medical Branch, Galveston, Texas, United States of America; 6 Research Laboratories, Toyama Chemical Company, Ltd., Toyama, Japan; 7 Sealy Center for Vaccine Development, The University of Texas Medical Branch, Galveston, Texas, United States of America; 8 Center for Biodefense and Emerging Infectious Diseases, The University of Texas Medical Branch, Galveston, Texas, United States of America; Aix Marseille University, Institute of Research for Development, and EHESP School of Public Health , France

## Abstract

**Background:**

Junín virus (JUNV), the etiologic agent of Argentine hemorrhagic fever (AHF), is classified by the NIAID and CDC as a Category A priority pathogen. Presently, antiviral therapy for AHF is limited to immune plasma, which is readily available only in the endemic regions of Argentina. T-705 (favipiravir) is a broadly active small molecule RNA-dependent RNA polymerase inhibitor presently in clinical evaluation for the treatment of influenza. We have previously reported on the *in vitro* activity of favipiravir against several strains of JUNV and other pathogenic New World arenaviruses.

**Methodology/Principal Findings:**

To evaluate the efficacy of favipiravir *in vivo*, guinea pigs were challenged with the pathogenic Romero strain of JUNV, and then treated twice daily for two weeks with oral or intraperitoneal (i.p.) favipiravir (300 mg/kg/day) starting 1–2 days post-infection. Although only 20% of animals treated orally with favipiravir survived the lethal challenge dose, those that succumbed survived considerably longer than guinea pigs treated with placebo. Consistent with pharmacokinetic analysis that showed greater plasma levels of favipiravir in animals dosed by i.p. injection, i.p. treatment resulted in a substantially higher level of protection (78% survival). Survival in guinea pigs treated with ribavirin was in the range of 33–40%. Favipiravir treatment resulted in undetectable levels of serum and tissue viral titers and prevented the prominent thrombocytopenia and leucopenia observed in placebo-treated animals during the acute phase of infection.

**Conclusions/Significance:**

The remarkable protection afforded by i.p. favipiravir intervention beginning 2 days after challenge is the highest ever reported for a small molecule antiviral in the difficult to treat guinea pig JUNV challenge model. These findings support the continued development of favipiravir as a promising antiviral against JUNV and other related arenaviruses.

## Author Summary

Argentine hemorrhagic fever (AHF) is a severe and often-fatal disease caused by infection with Junín virus (JUNV). Presently, there is an unmet need to develop new therapeutics to address current medical, public health and national security concerns, as JUNV is considered a potential bioterror agent amenable to aerosolization and intentional release. In the present study, favirpiravir, a promising anti-JUNV drug in clinical development for the treatment of influenza, was evaluated in an experimental small animal model of AHF. Guinea pigs challenged with JUNV were treated with favipiravir twice daily for two weeks starting 1–2 days after infection. Consistent with pharmacokinetic analysis that showed greater plasma levels of favipiravir in animals dosed by intraperitoneal injection, administration by this route resulted in a dramatic protective effect as 78% animals survived the infection compared to 11% in the placebo-treated group. Favipiravir treatment inhibited JUNV replication and prevented the development of disease observed in animals receiving placebo during the acute stage of infection. The high level efficacy observed following post-exposure prophylaxis with favipiravir is the highest ever reported for a small molecule antiviral in the guinea pig JUNV challenge model and thus supports its continued development as a promising antiviral therapy for the treatment of AHF.

## Introduction

Several New World (Junín, Machupo, Guanarito, Sabia, and Chapare) and Old World (Lassa and Lujo) arenaviruses cause viral hemorrhagic fever (HF) with case fatality rates generally in the range of 15–30% [1]. They are rodent-borne viruses that can be transmitted via the aerosol route therefore making them potential bioterror agents. Exposure to Lassa virus (LASV) and mortality associated with Lassa fever (LF) in hyperendemic areas of West Africa are estimated to be as high as 300,000 infections and 10,000 deaths annually [2]. Of the New World arenaviral HFs endemic in different regions of the South America, Junín virus (JUNV), the etiologic agent of Argentine HF (AHF), causes the greatest morbidity and mortality. AHF cases, although reduced in number, continue to be reported despite the vaccination of individuals with the greatest risk of exposure [3].

Immune plasma has proven to be an effective treatment for AHF if administered within 8 days of initial disease symptoms, but has been associated with a late neurological syndrome and is not readily available outside of Argentina [4]. Ribavirin is the only licensed antiviral known to offer protection in cases of LF [5], but remains largely unproven with only limited data in cases of AHF and Bolivian HF due to Machupo virus infection [6], [7]. Adverse effects primarily in the form of hemolytic anemia are generally considered to be reversible with cessation of ribavirin treatment [8]–[10]; however, teratogenicity and embryotoxicity are of concern [11], [12]. One must also consider the added risk of using a drug that can cause anemia to treat arenaviral HF diseases, which have a propensity for bleeding. At present, there are very few promising antivirals which have demonstrated anti-arenavirus activity *in vivo* [13].

Favipiravir (T-705; 6-fluoro-3-hydroxy-2-pyrazinecarboxamide) is a novel antiviral compound developed by the Toyama Chemical Co., which selectively and potently inhibits the RNA-dependent RNA polymerase (RdRP) of influenza [14]. It has been found to inhibit all serotypes and strains of influenza A, B and C viruses against which it has been tested [15], [16], including those resistant to currently approved neuraminidase inhibitors [17]. Remarkably, it is also active against alpha-, arena-, bunya-, and flaviviruses, both in cell culture and rodent models [16], [18]–[20], and it has shown *in vitro* activity against members of the paramyxo-, picorna-, and calicivirus families [21], [22].

To date, oral favipiravir treatment has been shown to be active in lethal hamster and guinea pig arenaviral HF models based on challenge with Pichindé virus (PICV), a New World arenavirus that produces in these species many of the clinical disease manifestations associated with AHF, including vascular leak and thrombocytopenia [23]–[25]. Importantly, favipiravir was efficacious in treating advanced disease even when initiating treatment one week after challenge when clinical disease was clearly apparent in guinea pigs [25]. *In vitro* studies have demonstrated favipiravir activity against pathogenic strains of JUNV, Machupo, and Guanarito viruses [19]; however, its activity against a bona-fide HF arenavirus *in vivo* has not been explored until now. In the present study, the antiviral activity of favipiravir against JUNV infection modeled in guinea pigs was investigated.

## Methods

### Ethics statement

All animal procedures complied with USDA guidelines and were conducted at the AAALAC-accredited Robert E. Shope, M.D. Laboratory at The University of Texas Medical Branch (UTMB; Galveston, TX) under protocol # 0903023 approved by the UTMB Institutional Animal Care and Use Committee.

### Animals

Outbred male Hartley strain guinea pigs (300–350 g) were obtained from Charles River (Wilmington, MA) and acclimated for 1 week prior to challenge. Animals were sorted prior to the start of both experiments so that the average group weights were similar. IPTT-300 electronic transponders were subcutaneously implanted for identification and temperature measurement in conjunction with the DAS 6002 scanner (BMDS, Seaford, DE).

### Virus

The Romero strain of JUNV was kindly provided by Thomas Ksiazek (UTMB). The virus stock was grown in Vero cells and the titer determined by plaque assay. The virus was prepared in minimal essential medium (MEM) for intraperitoneal (i.p.) challenge with approximately 1000 plaque-forming units (PFU). The actual challenge dose for each experiment was determined by plaque titration of the inoculation medium. All live virus work was performed in BSL-4 containment at UTMB in accordance with institutional health and safety standard operating procedures.

### Antiviral compounds

Favipiravir was provided by the Toyama Chemical Company, Ltd. (Toyama, Japan). Ribavirin was supplied by ICN Pharmaceuticals, Inc. (Costa Mesa, CA). Compounds were suspended in GERBER NatureSelect 1st FOODS carrot food (ingredients: carrots and water) for oral administration in the first efficacy study and 2.9% sodium bicarbonate solution (Sigma-Aldrich, St. Louis, MO) for i.p. dosing in the second study.

### Efficacy studies

#### Experiment 1

Groups of 10 guinea pigs each were treated orally twice daily approximately every 12 hours, for two weeks with approximately 300 mg/kg/day of favipiravir, 50 mg/kg/day ribavirin, or carrot baby food vehicle placebo starting 1 day post-infection (dpi) with 1300 plaque-forming units (PFU) of JUNV (determined by back titration of challenge medium). A 1 ml syringe was used to place the dose at the back of the palate for ingestion by the guinea pigs. Body weight and temperature were recorded on the mornings of the specified days during the study and morbidity and mortality were monitored daily for 40 days post-challenge. At 9 dpi, blood was collected by sampling from the cranial vena cava under deep isoflurane anesthesia. The sera were analyzed for viral titers as described below. Tissues (brain, liver and spleen) and serum were obtained from the surviving guinea pigs at the end of the study for JUNV-specific RT-PCR analysis, as well as histopathology.

#### Experiment 2

Groups of 12 guinea pigs each were treated i.p. with 300 mg/kg/day favipiravir, 50 mg/kg/day ribavirin, or 2.9% sodium bicarbonate vehicle placebo, divided into two daily doses (approximately every 12 hours) for 14 days beginning at 2 dpi with 750 PFU of JUNV (determined by back titration). On day 14, 3 pre-designated animals per group were sacrificed for hematology, blood chemistry, and virus titer analysis of serum, brain, heart, kidney, liver, lung, and spleen samples. The remaining 9 animals were monitored daily for morbidity and mortality and morning weights and temperatures were recorded on the indicated days.

### Serum and tissue virus titers

For virus titration of organs and serum collected from infected guinea pigs, approximately 0.2–0.5 g of each organ was homogenized in 0.5 ml PBS. Serum was separated from whole blood by centrifugation. The homogenates and sera were held at −80°C until titration. The homogenates were centrifuged to remove cellular debris and the cleared homogenate for each organ and the serum were titrated by a focus-forming unit (FFU) assay as follows.

Vero E6 monolayers were infected with serial 10-fold dilutions of serum or tissue homogenate for one hour at 37°C. For the second experiment, serum was also tested undiluted. Following infection, cells were overlayed with 0.8% Tragacanth (Sigma-Aldrich) in MEM supplemented with 2% fetal bovine serum and 1% penicillin and streptomycin. After 7 days in culture, the overlay was removed, the cells fixed with 10% buffered formalin for 30 min at room temperature followed by overnight refrigeration. Fixed cells were permeabilized in 70% ethanol for 20 min and washed with phosphate buffered saline (PBS) prior to overnight staining with primary antibody (antisera to JUNV Candid #1 kindly provided by Dr. Robert Tesh, World Reference Collection for Emerging Viruses and Arboviruses, UTMB) diluted 1∶1000 in PBS with 5% milk and 1% Tween 20. The cells were then washed with PBS and the secondary antibody, goat anti-mouse IgG labeled with horseradish peroxidase (HRP; DakoCytomation, Carpinteria, CA) diluted 1∶500 in PBS with 1% bovine growth serum (BGS), was added to plates and incubated for 4–5 h. After washing with PBS, AEC Substrate Chromagen (DakoCytomation) was added for 15 min and the reaction was stopped with distilled water prior to counting of FFUs.

### Plaque reduction neutralization titer (PRNT) assays

Heat-inactivated guinea pig sera from survivors were diluted 1∶10 in MEM supplemented with 1% FBS, and titrated in two-fold serial dilution steps. Equal volumes (150 µl) of JUNV Romero strain containing approximately 1000 PFU/mL and serum dilutions were mixed and incubated for 1 h at 37°C and 5% CO_2_. Confluent monolayers of Vero E6 cells (seeded in 12-well plates) were infected with 100 µl of the virus–serum mixtures. After 1 h incubation at 37°C and 5% CO_2_, the wells were overlaid with 0.5% agarose MEM with 1% FBS. The plates were incubated at 37°C and 5% CO_2_ for 7 days, and then stained with 0.25% crystal violet in 10% buffered formalin. The plates were washed and the plaques enumerated. The neutralizing antibody titer of a serum was considered positive at the highest initial serum dilution that resulted in >80% (PRNT_80_) reduction of the number of plaques as compared to guinea pig serum from the mock-infection.

### Hematology and blood chemistry

All cell counts were quantified using a Hemavet Mascot hematology analyzer (Drew Scientific, Dallas, TX) equipped with veterinary software to measure white blood cell count, red blood cell count, hemoglobin concentration, hematocrit, mean corpuscular volume, mean corpuscular hemoglobin, mean corpuscular hemoglobin concentration, neutrophils, lymphocytes, monocytes, eosinophils, basophils, leucocytes, reticulocytes, and platelet count. Blood chemistry was performed using a VetScan2 Chemistry Analyzer (Abaxis, Inc., Sunnyvale, CA), which provides a diagnostic panel that includes albumin, alkaline phosphatase, alanine aminotransferase, amylase, total bilirubin, blood urea nitrogen, calcium, creatinine, glucose, potassium, total protein and globulin.

### Pharmacokinetic analysis

Guinea pigs were treated with 100 mg/kg of favipiravir administered by placement of drug prepared in carrot baby food vehicle in the back of the oral cavity with a tuberculin syringe or by i.p. injection of drug prepared in a 2.9% sodium bicarbonate solution. Plasma was obtained from 3 animals per group by saphenous vein puncture at 0.25, 0.5, 1, 2 or 4 h after treatment. Samples were deproteinized and analyzed by HPLC for quantitation of favipiravir as previously described [25].

### Statistical analysis

The Mantel-Cox log-rank test was performed to analyze Kaplan-Meier survival plots. Hematology and blood chemistry were analyzed by the Student's two-tailed t-test. Virus titers were analyzed using one-way analysis of variance (ANOVA) followed by Bonferroni multiple comparisons test. All statistical evaluations were done using Prism (GraphPad Software).

## Results

### Evaluation of favipiravir in the guinea pig JUNV infection model: Oral dosing trial

**Figure 1:**
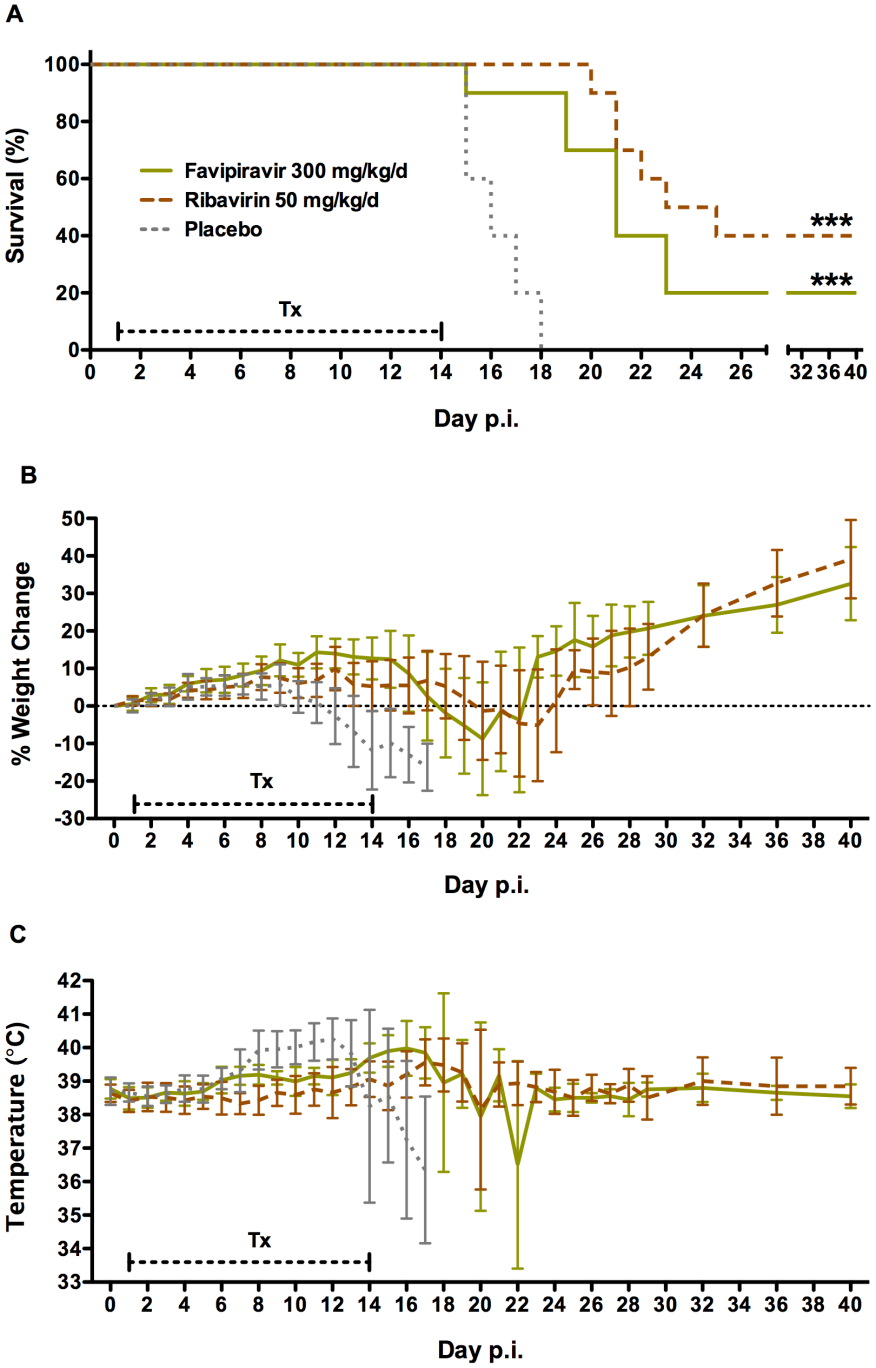
Survival outcome following oral treatment of JUNV-infected guinea pigs with favipiravir. Guinea pigs (n = 10/group) were challenged i.p. with 1300 PFU of JUNV. They were dosed by instillation of favipiravir, ribavirin, or carrot baby food vehicle (placebo) into the back of the oral cavity. Treatments (Tx) with the indicated concentrations of drugs were initiated 24 h post-infection (p.i.) and administered twice daily for 14 days (capped hashed line). A) survival, B) mean body weight (relative to initial starting weight), and C) temperature were monitored for 40 days. ***P<0.001 compared to placebo-treated animals by the log-rank test.

Based on studies modeling oral favipiravir therapy in PICV-infected guinea pigs [25], a dose of 300 mg/kg/day was selected for the initial efficacy trial in the JUNV infection model. Animals were dosed twice daily by oral installation for a duration of 14 days, starting 1 day after challenge with 1300 PFU of the Romero strain of JUNV. As shown in Figure 1A, favipiravir treatment improved survival outcome compared to guinea pigs treated with placebo. Two of the 10 guinea pigs treated with favipiravir survived the infection, and those that succumbed survived, on average, >4 days longer than the animals treated with placebo (20.3±2.6 days and 16.2±1.2 days, respectively).

Weight change over the course of the study was used to assess the effect of favipiravir treatment on the condition of the animals. The mean weights of the placebo-treated guinea pigs began to drop on day 10 post-infection, and were below the initial starting weights by day 12 (Figure 1B). In contrast, mean weights in animals treated with favipiravir did not fall below initial starting weights until day 18. Favipiravir treatment also delayed the onset of fever (defined as a body temperature of ≥39.8°C) from 8.8±2.3 days in the placebo group to 16.5±3.3 days. Ribavirin, included as a positive control [26], performed similarly to favipiravir as the survival, weight, and temperature curves did not differ significantly between the two drug treatment groups.

Blood samples were collected from all animals on day 9 post-infection for analysis of virus titers. The early time point was selected because of concerns that the deep anesthesia required for blood collection from the cranial vena cava may have resulted in the loss of sick animals if the procedure would have been delayed beyond day 9. Unfortunately, viral titers in the placebo-treated animals were not well developed, as only 3 of 10 animals had detectable levels of virus in the range of 200–1300 FFU/ml. Nevertheless, virus could not be detected in favipiravir- or ribavirin-treated guinea pigs. Viral RNA was not detected in the serum or brain, liver or spleen tissues collected from the surviving favipiravir- and ribavirin-treated animals at the conclusion of the study (data not shown). Moreover all survivors appeared to be in good health by physical examination and all the aforementioned tissues were histologically normal.

### Comparison of favipiravir PK in guinea pigs treated by oral and i.p. routes

**Figure 2:**
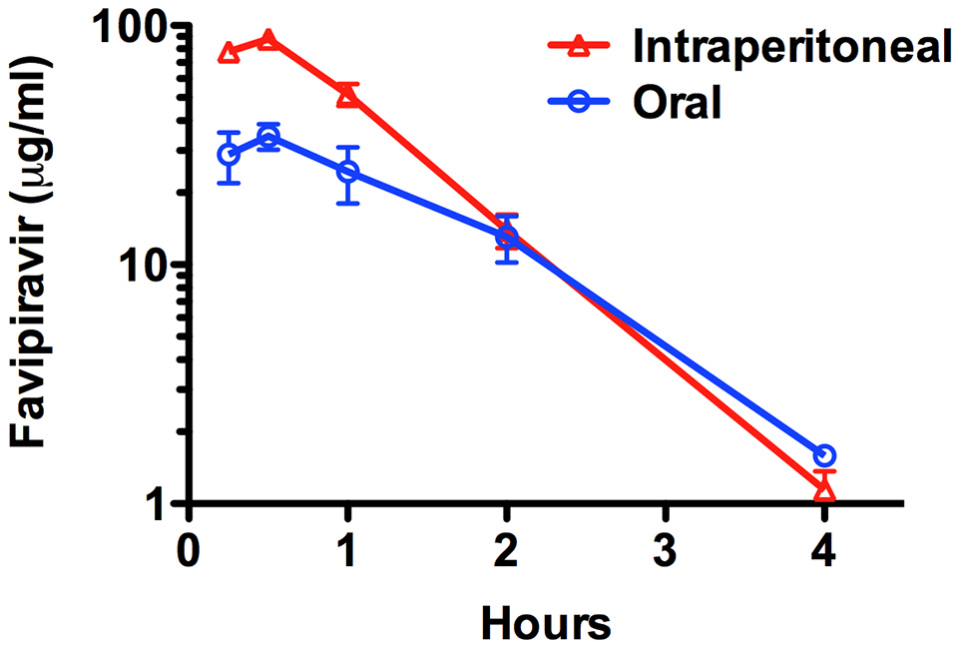
PK analysis of favipiravir in male Hartley guinea pigs dosed by oral instillation or intraperitoneal (i.p.) injection. Favipiravir (100 mg/kg) was administered orally in carrot baby food vehicle or by i.p. injection in 2.9% sodium bicarbonate. Longitudinal plasma favipiravir levels are shown from 3 animals per treatment group at 15 and 30 minutes, and 1, 2, and 4 h after treatment. Data points represent the mean and standard error of the mean.

Despite proven effectiveness in the PICV guinea pig infection model by oral treatment with favipiravir suspended in carrot baby food vehicle [25], the lower than expected efficacy observed in the first study prompted a PK analysis to compare plasma levels of favipiravir following administration by i.p. and oral treatments. Compared to oral instillation of the compound, i.p. injection of an equivalent dose of 100 mg/kg resulted in higher plasma concentrations of favipiravir within the first hour, and similar levels at the later time points (Figure 2). The area under the curves (AUC)s of the drug concentrations were 56.1 by the oral instillation method and 103.4 by i.p. injection. Peak favipiravir plasma concentrations approximate 80 µg/ml (500 µM) for i.p. treatment, with >10 µg/ml (60 µM) present at 2 h. These levels are well above the reported 50% effective concentration (EC_50_) of <20 µM for JUNV in cell culture [19]. Thus, the single dose PK data suggest that favipiravir is more bioavailable through i.p. dosing.

### Evaluation of favipiravir in the guinea pig JUNV infection model: i.p. dosing trial

**Figure 3:**
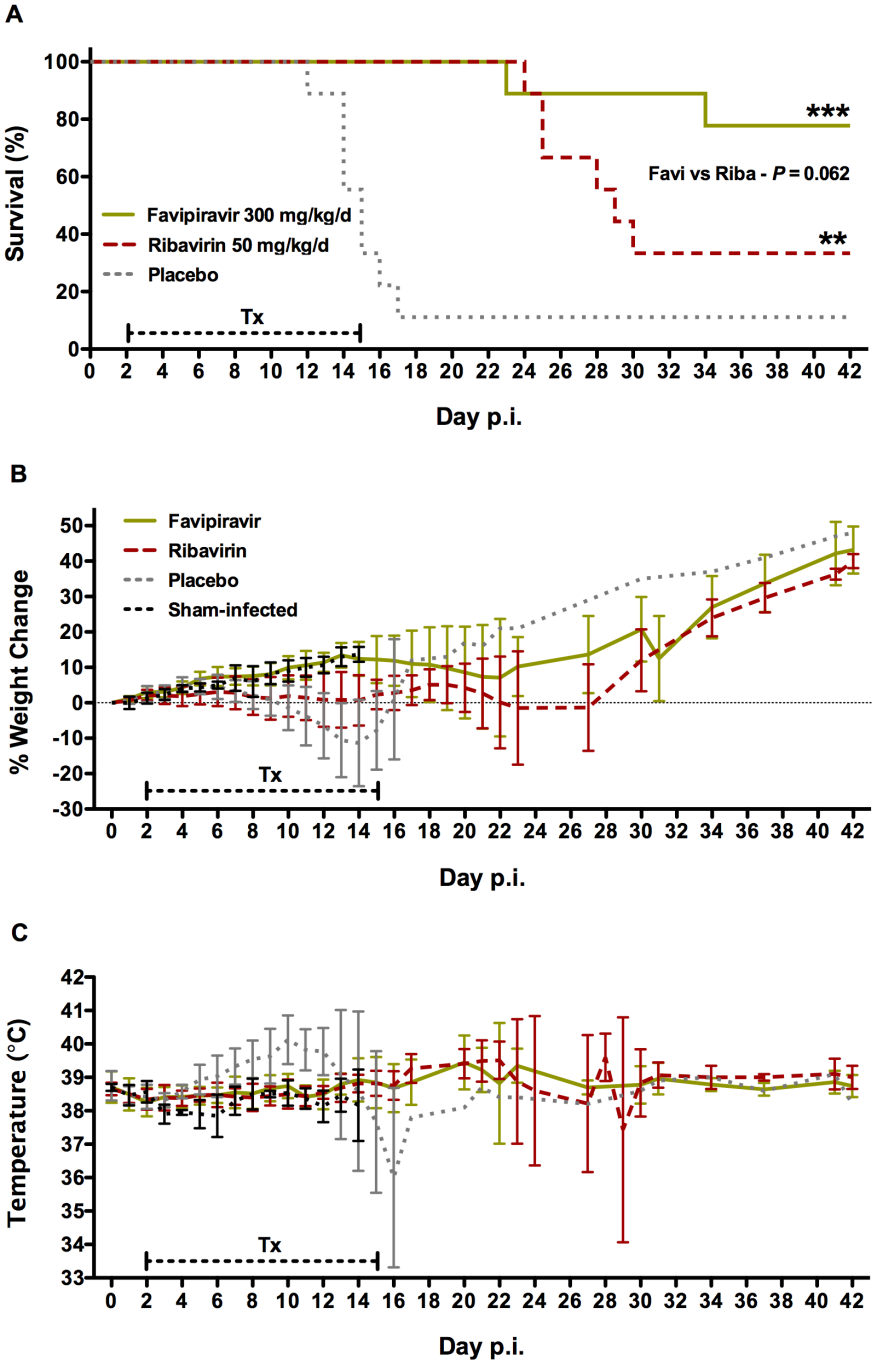
Survival outcome following i.p. treatment of JUNV-infected guinea pigs with favipiravir. Guinea pigs (n = 12/experimental group) were challenged i.p. with 750 PFU of JUNV and dosed i.p. with favipiravir (300 mg/kg/d), ribavirin (50 mg/kg/d), or 2.9% sodium bicarbonate vehicle (placebo) beginning 48 h post-infection (p.i.). Treatments (Tx) were administered twice daily for 14 days (capped hashed line). A) survival (n = 9/group), and B) mean body weight (relative to initial starting weight) and C) temperature were monitored in all surviving animals for 42 days. **P<0.01, ***P<0.001 compared to placebo-treated animals by the log-rank test.

A second experiment was conducted wherein favipiravir was dosed by i.p. injection starting 48 h post-challenge with 750 PFU of JUNV. Favipiravir treatment started 2 days post-infection provided a highly significant level of protection (7/9 survivors; 78%) compared to the placebo group (1/9 survivors; 11%), and performed better than ribavirin (3/9 survivors; 33%) (Figure 3A). The weight data were consistent with the survival curves with the favipiravir-treated animals mirroring the weight gain of the sham-infected animals through day 14 before leveling out temporarily as a few animals became ill (Figure 3B). Guinea pigs in the placebo group began to develop fevers as early as day 6 post-infection, while temperatures were in the normal range in the drug-treated animals through the first two weeks (Figure 3C). Notably, the surviving placebo-treated animal had undetectable (PRNT_80_<20) levels of JUNV neutralizing antibodies at the conclusion of the 42-day study. In contrast, all guinea pigs treated with favipiravir or ribavirin developed substantial neutralizing antibody titers of 320. The placebo group survivor also gained weight and maintained normal body temperature throughout the entire experiment suggesting that the virus challenge did not produce the desired infection.

**Figure 4:**
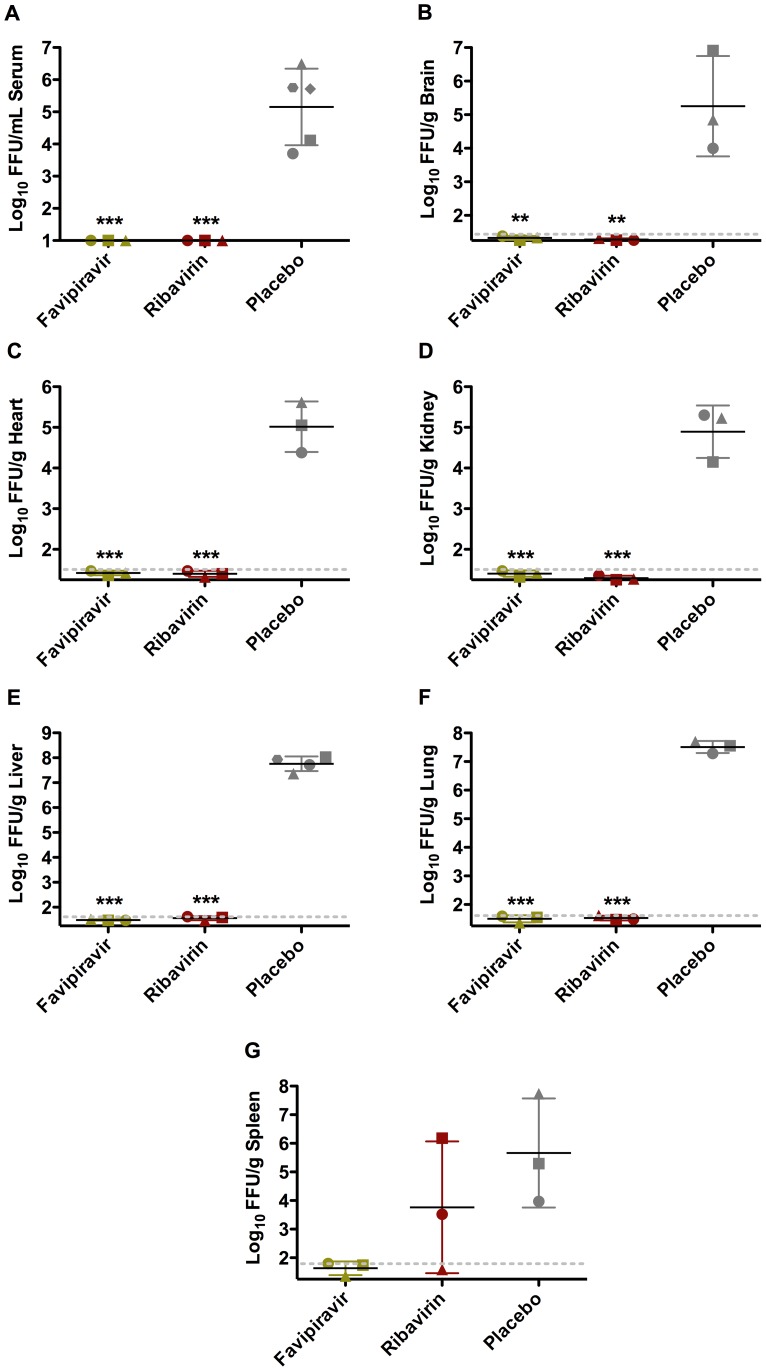
Effect of i.p. favipiravir treatment on day 14 viral loads in JUNV-challenged guinea pigs. Animals were infected and treated as described in Figure 3. Three pre-designated animals in each treatment group were sacrificed on day 14 post-infection for analysis of A) serum, B) brain, C) heart, D) kidney, E) liver, F) lung and G) spleen virus titers. Two serum samples and 1 liver sample collected from 2 moribund animals from the placebo group euthanized on days 12 and 14 were also included in the analysis. Unique symbols in each treatment group represent values for the same animal across all parameters and hashed lines indicate the assay limits of detection in tissue samples. **P<0.01, ***P<0.001 compared to placebo.

In a subset of animals sacrificed on day 14 of infection, favipiravir treatment prevented the thrombocytopenia and leucopenia commonly associated with severe disease and also maintained a number of hematologic and blood chemistry parameters at normal baseline levels (Table 1). Moreover, we were unable to detect virus in the serum, brain, heart, kidney, liver, lung, and spleen of favipiravir-treated animals (Figure 4). With the exception of a single animal that had >5 logs of virus per gram of spleen, guinea pigs treated with ribavirin were also free of virus. All surviving guinea pigs were rapidly gaining weight and observed to be in good health by physical examination at the termination of the study. Taken together, the results demonstrate robust inhibition of viral replication and a high level of protection by i.p favipiravir treatment in the difficult to treat JUNV guinea pig infection model.

**Table 1:** Hematology and blood chemistry analysis on day 14 post-infection in the i.p. treatment efficacy study^a^.

Blood Component^b^ (units)	Normal	Placebo	Ribavirin	Favipiravir
**Hematology**				
WBC (K/µL)	3.68±1.85*	0.82±0.21	2.59±0.92*	5.61±1.86*
Neu (K/µL)	2.04±1.30	0.48±0.30	2.07±0.69*	3.11±1.90
Neu (%)	53.5±8.3	52.8±33.1	80.3±2.1^d^	52.6±15.1
Lym (K/µL)	1.57±0.64*	0.29±0.14	0.46±0.23^c^	2.40±0.58**
Lym (%)	44.1±8.3	40.9±31.4	17.1±3.4^d^	45.5±14.6
Mon (K/µL)	0.05±0.04	0.03±0.02	0.01±0.01	0.08±0.01*
Mon (%)	2.08±2.07	3.35±2.45	0.39±0.40	1.53±0.60
Eos (K/µL)	0.02±0.02	0.01±0.02	0.06±0.07	0.02±0.01
Eos (%)	0.34±0.37	2.20±2.80	2.15±2.59	0.38±0.29
Bas (K/µL)	0.00±0.00	0.01±0.01	0.00±0.00	0.00±0.00
Bas (%)	0.02±0.03	0.72±0.70	0.01±0.02	0.04±0.05
RBC (M/µL)	5.59±0.20	3.86±1.67	5.62±0.27	5.92±0.08
Hb (g/dL)	13.9±0.6	9.30±4.3	13.5±0.6	14.4±0.4
HCT (%)	48.4±2.5	31.4±13.7	46.4±3.0	50.7±0.9
MCV (fL)	86.7±2.5*	81.1±0.7	82.5±1.5	85.7±0.7**
MCH (pg)	24.8±0.6	23.9±1.0	24.1±0.1	24.2±0.3
MCHC (g/dL)	28.7±0.2	29.4±1.0	29.2±0.6	28.3±0.2
RDW (%)	13.9±0.9	14.0±0.7	14.0±1.0	14.2±0.4
PLT (K/µL)	476±63***	27±16	401±97**	554±120**
MPV (fL)	6.03±0.99	8.15±1.06	5.47±0.25*	6.50±0.53
**Blood Chemistry**				
ALB (g/dL)	3.97±0.40*	2.23±0.83	3.57±0.42	3.73±0.06*
ALP (U/L)	240±34	334±326	119±44^c^	187±37
ALT (U/L)	54±14	109±47	81±27	43±4
AMY (U/L)	1320±198	1503±326	1559±593	1241±54
TBIL (mg/dL)	0.30±0.00	0.30±0.00	0.30±0.00	0.33±0.06
BUN (mg/dL)	13.7±0.6*	35.7±12.5	14.3±3.2*	13.3±1.2*
Ca (mg/dL)	11.3±0.2***	9.0±0.2	10.8±0.7*	11.3±0.2***
PHOS (mg/dL)	4.73±0.45	8.10±5.72	3.73±1.06	5.60±0.75
CRE (mg/dL)	0.20±0.00	0.20±0.00	0.20±0.00	0.27±0.12
GLU (mg/dL)	185±22	212±37	138±9c, *	167±4
Na^+^ (mM)	138±2	140±3	134±2*	136±2
K^+^ (mM)	4.47±0.31	4.30±1.39	5.23±0.78	4.93±0.45
TP (g/dL)	4.97±0.06	4.17±0.49	4.37±0.31^c^	4.77±0.12
GLOB (g/dL)	0.97±0.29*	1.90±0.30	0.80±0.20**	1.07±0.12*

= 3/group) treated twice daily with 300 mg/kg/day favipiravir, 50 mg/kg/day ribavirin, or placebo starting 48 h post-challenge with 750 PFU of JUNV were sacrificed on day 14 of infection for sample collection. Whole blood and sera were analyzed for hematology and blood chemistry, respectively.^a^ Guinea pigs (n

^+^, sodium; K^+^, potassium; TP, total protein; GLOB, globulin.^b^ WBC, white blood cells; Neu, neutrophils; Lym, lymphocytes; Mon, monocytes; Eos, eosinophils; Bas, basophils; RBC, red blood cells; Hb, hemoglobin; HCT, hematocrit; MCV, mean corpuscular volume; MCH, mean corpuscular hemoglobin; MCHC, mean corpuscular hemoglogin concentration; RDW, red cell distribution width; PLT, platelets; MPV, mean platelet volume; ALB, albumin; ALP, alkaline phosphatase; ALT, alanine aminotransferase; AMY, amylase; TBIL, total bilirubin; BUN, blood urea nitrogen; Ca, calcium, PHOS, phosphate; CRE, creatinine; GLU, glucose; Na

*P*<0.05,^c^

d *P*<0.01, compared to mock-infected normal controls by the Student's two-tailed t-test.

* *P*<0.05,

** *P*<0.01,

*** *P*<0.001 compared to placebo by the Student's two-tailed t-test.

## Discussion

In the present study, the efficacy of oral and i.p. favipiravir treatment was evaluated in the guinea pig JUNV challenge model. Based on previous success in treating PICV infection in guinea pigs [25], favipiravir was initially dosed by orally feeding the animals the drug suspended in carrot baby food. The results from this trial did demonstrate a significant protective effect that was comparable to ribavirin, the only small molecule antiviral that has demonstrated activity against severe JUNV infection, which initially showed promise over 25 years ago [27], [28]. Ribavirin has been evaluated in a small-scale clinical trial in patients with advanced cases of Argentine HF, and did produce some signs of efficacy [3]; however, because it is associated with toxicity primarily in the form of hemolytic anemia, safer and more effective options are needed. The results from the initial guinea pig study were encouraging and supported further consideration of favipiravir through a second experiment designed to improve upon the limited success of the first study.

The fact that oral favipiravir provided complete protection against lethal PICV infection in guinea pigs underscores the greater challenge of treating the more virulent JUNV infection, which was also less responsive to ribavirin in the present and past studies [25]–[27]. It is likely that the higher plasma concentrations achieved when administering favipiravir by the i.p. route resulted in greater levels of drug in target organs, which may have contributed to the remarkable efficacy observed in the second experiment. Because the animals generally did not like the taste of favipiravir suspended in the baby food vehicle, it was difficult to accurately deliver the doses in BSL-4 containment. This may have led to diminished amounts of drug actually making it into the gut for subsequent absorption into the circulation.

It is likely that higher doses of the well-tolerated oral favipiravir [25] would improve survival outcome in guinea pigs challenged with JUNV; however, the increased viscosity of the favipiravir suspension at higher drug concentrations results in a paste-like consistency that is increasingly difficult to administer by mouth. It is also possible that delaying the initiation of treatment from 24 h in the first study to 48 h for the second study may have elicited a better immune response to the additional 24 h of viral replication, which combined with the inhibitory effects of favipiravir may have facilitated the clearance of the virus and afforded the greater level of protection observed. Notably, the clinical laboratory findings and lack of virus on day 14 correlated well with the survival data.

The principal mechanism of action of favipiravir against influenza A virus was shown to be direct inhibition of the viral polymerase [14]. Although direct evidence to support this claim is lacking for other viruses sensitive to the action of favipiravir, findings from arenavirus and norovirus studies are consistent with the RdRP serving as the main target [19], [21]. A recent report suggests that favipiravir induces lethal mutagenesis in influenza A viruses through selective pressure applied in cell culture [21]. However, it is uncertain whether this mechanism plays any role *in vivo*. Collectively, the evidence suggests that favipiravir selectively inhibits RNA virus RdRP, with only limited toxicity to cells. This specificity makes favipiravir an attractive candidate for a broadly active therapeutic with potential to treat multiple viral diseases. Our findings with i.p. favipiravir treatment represent the most significant level of protection ever reported for an antiviral drug intervention in the difficult to treat JUNV guinea pig infection model [26]. A study to define the therapeutic window in guinea pigs and efficacy studies in a nonhuman primate model are planned.
